# Effect of Cognition Recovery by Repetitive Transcranial Magnetic Stimulation on Ipsilesional Dorsolateral Prefrontal Cortex in Subacute Stroke Patients

**DOI:** 10.3389/fneur.2022.823108

**Published:** 2022-01-31

**Authors:** Jongwook Kim, Byoungwoo Cha, Doyoung Lee, Jong Moon Kim, MinYoung Kim

**Affiliations:** ^1^Department of Rehabilitation Medicine, CHA Bundang Medical Center, CHA University School of Medicine, Seongnam, South Korea; ^2^Rehabilitation and Regeneration Research Center, CHA University School of Medicine, Seongnam, South Korea

**Keywords:** stroke, repetitive transcranial magnetic stimulation, cognition, DLPFC (dorsolateral prefrontal cortex), neurorehabilitation, ipsilesional dorsolateral prefrontal cortex, subacute stroke

## Abstract

**Objective:**

To demonstrate the efficacy of high-frequency repetitive transcranial magnetic stimulation (rTMS) over the ipsilesional dorsolateral prefrontal cortex (DLPFC) on neurological recovery in patients with subacute phase stroke.

**Methods:**

Patients with supratentorial hemispheric stroke who were hospitalized for intensive rehabilitation in the subacute phase were enrolled for this retrospective analysis. Two groups of patients were selected: the rTMS group who received high-frequency (20 Hz) rTMS ≥ 5 times over the ipsilesional DLPFC, and a control group who did not receive any rTMS. The patients were further divided into groups with right- or left-side brain lesions. Functional measurements for cognitive ability, mood, speech, and activities of daily living, which were assessed at baseline and at the 1-month follow-up as a routine clinical practice, were used for analyses.

**Results:**

Among 270 patients with available clinical data, 133 (women, 51; age, 61.0 ± 13.8 years) met the inclusion criteria and were enrolled for analysis. There were no differences in demographic data and functional scores at baseline between the rTMS (*n* = 49) and control (*n* = 84) groups. The rTMS group showed a higher gain in the mini-mental status examination (MMSE) total score and subscores of all domains, forward digit span, and FIM-cognition than the control group (*P* < 0.05). Among the patients with left hemispheric lesions (*n* = 57), the rTMS group showed better outcomes in cognition and depression through scores of total and “attention and concentration” subscores of MMSE, FIM-cognition, and the geriatric depression scale (*P* < 0.05). Among the patients with right hemispheric lesions (*n* = 76), the rTMS group showed better outcomes in cognition through the MMSE total score and subscores of “attention and concentration,” “registration,” and “recall,” and scores of both forward and backward digit spans (*P* < 0.05).

**Conclusion:**

High-frequency rTMS over the ipsilesional DLPFC has beneficial effects on the recovery of cognition on both sides as well as mood in patients with left-sided hemispheric lesions.

## Introduction

Cognitive impairment and depression are common complications after stroke (1) which lead to slow recovery in activities of daily living (ADL) (2). Although various rehabilitation techniques and medications are constantly being attempted, their therapeutic effects have been limited (3). Repetitive transcranial magnetic stimulation (rTMS) has emerged as an alternative therapeutic avenue for neurological recovery from stroke sequelae (4) and its beneficial effects are becoming increasingly evident (5). It has been demonstrated that rTMS activates neural plasticity by shifting synaptic weighting, sprouting new dendritic connections, and forming new synapses (6). Adaptive neural plasticity induced by rTMS, including changes in synaptic connectivity and excitability in surviving neural cells in lesions and peri-lesions, is directly related to functional recovery in patients with stroke (7, 8). Previous research revealed temporary recovery from post-stroke cognitive impairment by applying rTMS to the left dorsolateral prefrontal cortex (DLPFC) (9) and various attempts have already proven its effect on psychiatric symptoms in patients with completed stroke (5, 10, 11). In particular, rTMS therapy on the left DLPFC was approved for the treatment of depression in USA in 2008 (12) and has been widely used for treatment-resistant depression in many countries (13).

It is well known that the DLPFC in the bilateral hemisphere plays a key role in various cognitive processes, such as attention, working memory, cognitive flexibility, planning, inhibition, and abstract reasoning (14, 15). However, the precise role of each DLPFC in cognition has not been clearly identified (16). Many previous therapeutic approaches for patients with stroke have focused on the left DLPFC, mostly when it was proposed to ameliorate cognitive impairment and depression (17–19). Treatments with rTMS over the left DLPFC has been reported to enhance working memory and executive functioning in patients with stroke (20, 21). However, clinical studies investigating the effects of rTMS over the right DLPFC are relatively insufficient, and there is still controversy regarding the role of the right DLPFC (22) and transcallosal connections between the left and right DLPFC (23). Meanwhile, there are reports that indicate involvement of the right DLPFC in the retrieval of information from episodic memory (24, 25).

According to our clinical experience of rTMS application in several ways for cognitive enhancement, high-frequency stimulation of the ipsilesional DLPFC was effective in patients with supratentorial hemispheric stroke. Functional and structural studies have indicated that the DLPFC is connected to a variety of brain areas, including the thalamus, basal ganglia, and primary and secondary association areas of the neocortex, including the posterior temporal and parietal areas (26) and the connectivity was significantly correlated with the corresponding cognitive performance (27). To the best of our knowledge, no studies have evaluated the therapeutic efficacy of ipsilesional rTMS. Moreover, no reports have compared the effects on cognition according to the applied hemispheric side of the DLPFC, left *vs*. right. Therefore, we conducted a retrospective study investigating the effect of high-frequency rTMS on cognition when it was applied to the ipsilesional DLPFC and analyzed the effect on each side of the lesion in patients with supratentorial hemispheric stroke during the subacute phase.

## Materials and Methods

### Patients

**Figure 1:**
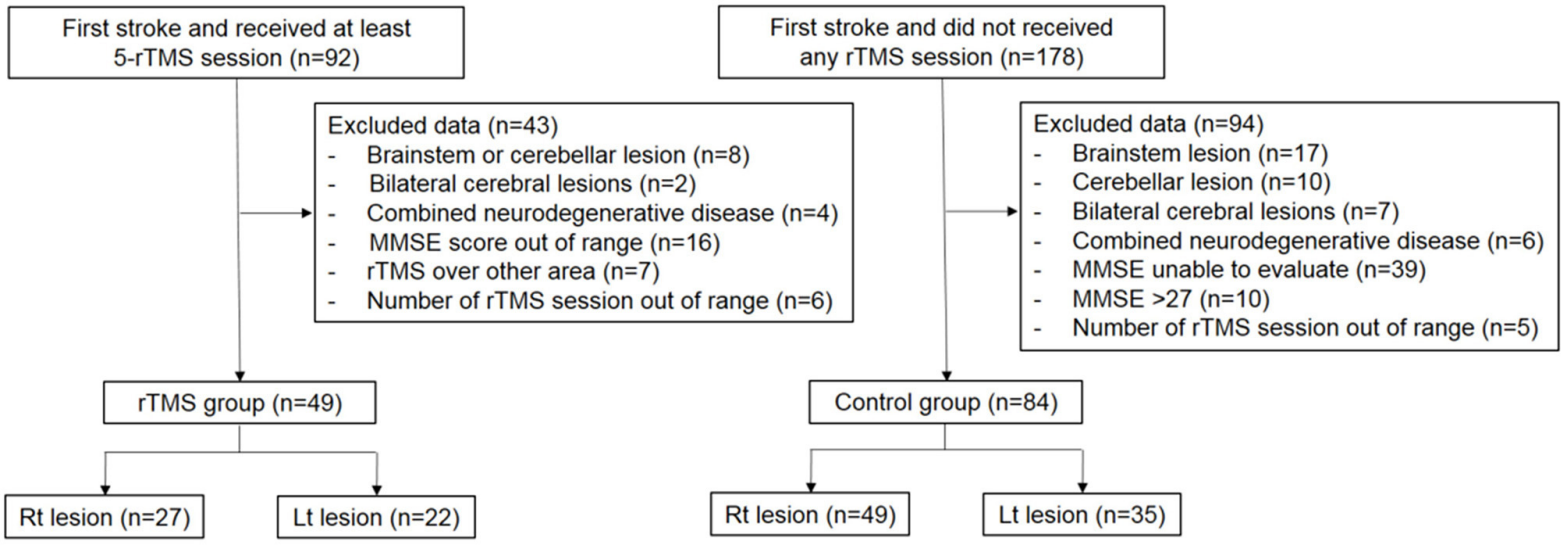
Flow chart of subject selection for analyse. rTMS, repetitive Transcranial Magnetic Stimulation; MMSE, Mini-Mental State Examination.

This retrospective analysis was conducted at the rehabilitation center of a university-affiliated general hospital in charge of intensive stroke rehabilitation immediately after being stabilized from acute care. The study was approved by the institutional review board of the study hospital (IRB file No: 2017-09-060 and 2021-12-039). All the corresponding patients were enrolled without exception according to the following inclusion criteria: (1) subacute period of the first ever completed stroke, 15–90 days after the onset; (2) unilateral supratentorial hemispheric stroke lesion; and (3) over 18 years of age who were admitted and received intensive rehabilitation for at least 4 weeks between March 2014 and December 2019 (Figure 1). Exclusion criteria were: (1) brainstem or cerebellar lesion, (2) involvement of the lesion in the contralateral cerebral hemisphere, (3) signs suggestive of degenerative neurological diseases such as Parkinson's disease or Alzheimer's disease, (4) patients with severe cognitive impairment who were incapable of assessment with the Mini-Mental Status Examination (MMSE) score, (5) no remarkable impairment of cognition with MMSE score > 26 points, and (6) patients who received rTMS over cortices other than the DLPFC or received rTMS 1–4 times.

The rTMS treatment was performed at least three times a week for four consecutive weeks for each patient. According to a meta-analysis that referred 5–10 times of high-frequency rTMS on the DLPFC as a therapeutic intervention (5), this study analyzed the therapeutic effect of rTMS by comparing outcomes in two groups of patients: rTMS-treated group, who received ≥ 5 times of rTMS treatment, and the control group, who never received rTMS.

All the patients in this study received intensive rehabilitation consisting of 30–40 min of physical therapy twice and 30–40 min of occupational therapy per day, 5 days a week for weekdays, and once on weekends. While physical therapy targets gross motor recovery, occupational therapy aims to enhance various cognitive abilities, facilitates the use of disabled upper extremities, and promotes independent ADL. Although an individualized therapeutic approach was applied to each patient, the overall framework was based on the standardized protocol of the trained rehabilitation team of the university hospital (28). And occupational therapy was implemented as soon as rTMS was finished (in a 10 mins), with the exception of a few due to urgent events.

### Repetitive Transcranial Magnetic Stimulation

The rehabilitation center has provided rTMS as an optional therapy to facilitate the rehabilitation of patients. A patient or legal guardian may choose the therapy on their own after being informed by the physician about rTMS.

Stimulation was performed using a biphasic stimulator (MagPro® Dantec, Denmark; from March 2014 to May 2018 and ALTMS® Remed, Republic of Korea; from May 2018 to December 2019) and a 70-mm figure-eight coil. The routine stimulation procedure is described as follows. First, the resting motor threshold was measured for each patient. At the lowest stimulus output, a stimulus was applied to the M1 cortex of the uninjured hemisphere aiming at the site that caused the largest visible twitch in the participant's thumb. The resting motor threshold was defined as the intensity required to generate a motor-induced potential of > 50 μV in the contralateral abductor pollicis brevis muscle at least 5 out of 10 times with 30 s of inter-stimulus time interval (29, 30).

According to the prescription, rTMS was administered by trained physicians over the ipsilesional DLPFC, which approximately corresponds to Brodmann area 9/46, 5 cm forward along the parasagittal line at the M1 cortex location where the abductor pollicis brevis muscle was activated. The angle of the coil was inclined 45° relative to the sagittal line of the head (18, 31). Stimulation was given at 80% of the resting motor threshold stimulator output: 20 Hz, 5-s train duration, 55-s inter-train interval, and a total number of stimulations of 1,000 pulses per session for 20 min (5). Any side effects were monitored during rTMS treatment and up to 10 min after treatment in the therapy room.

### Assessment of Outcome and Side Effects

All data were obtained from electronic medical records according to the medical practices in the study hospital and evaluated at admission within a week, followed by the same assessments 4 weeks after completing the initial evaluations as a routine process. For appropriate rehabilitation, the patients underwent assessments for cognitive, affective, and language abilities, including MMSE (32), Wechsler Adult Intelligence Scale-IV (WAIS-IV) (33), forward and backward digit spans (34), mood evaluation using the Geriatric Depression Scale (GDS) (35), language evaluation with aphasia quotient (AQ) by Western Aphasia Battery (WAB) (36). Aphasia diagnosis and classification were made by a standardized evaluation criteria varied depending on gender, age, educational level (37). And unilateral visual neglect evaluated by both line bisection test (38) and Albert test (39) which have been used globally as screening tools. The total MMSE score and its subscore for each domain, orientation, registration, recall, attention/concentration, and language (32, 40, 41), were used for retrospective analysis, which have been utilized to measure cognition in patients with stroke (42, 43). Intelligence quotient (IQ) was measured with WAIS-IV, digit span score was evaluated with numbers, and AQ percentile was measured with WAB. All assessments were conducted using nationality appropriate and authorized versions by a clinical psychologist and two speech therapists who were expert professionals. The functional independence measure (FIM) (44) was used to evaluate performance ability in ADL. The FIM cognition scale is composed of five cognitive items assessing the abilities of communication and social cognition with a range of 5–35 points. These assessments were conducted by occupational therapists who were trained and passed the reliability test.

Adverse events were defined according to the Common Terminology Criteria for Adverse Events (CTCAE) Version 4.0 (45). The side effects were evaluated in all patients who received rTMS, regardless of group designation. Since the treatment was given by a physician, adverse events were directly monitored during the procedure, and the patients were closely monitored during their hospitalization period by the attending staff.

### Statistical Analysis

The Statistical Package for the Social Sciences version 21.0 for Windows (SPSS Inc., Chicago, IL, USA) was used for data analyses. The independent *t*-test was used to compare the demographic data and baseline characteristics between the rTMS and control groups and to compare the outcomes (i.e., score changes from baseline to follow-up) between both groups. A paired *t*-test was used to confirm the changes in scores before and after treatment within the group. Normality was verified based on the results of the Kolmogorov-Smirnov test for the difference between baseline and follow-up in the measured scores. Statistical significance was set at *P* < 0.05.

## Results

### Overall Patient Characteristics

Data were collected from 270 patients who underwent initial assessment and follow-up assessments after 4 weeks of rehabilitation. Finally, 49 patients met the inclusion criteria for the rTMS-treated group by receiving rTMS ≥ five times on the ipsilesional DLPFC, and 84 patients were included in the control group (Figure 1). The mean age of the rTMS-treated group was 60.4 ± 14.4 years (42.8% female), and that of the control group was 61.3 ± 13.5 years (35.7% female) without differences in demographic characteristics between the groups regarding age, sex, type of stroke, and post-stroke duration. The patients in the rTMS-treated group received 8.7 ± 2.4 times (right hemispheric stroke: 9.1 ± 2.2, left hemispheric stroke: 8.0 ± 2.6) of rTMS.

The baseline characteristics of the rTMS-treated group and control group did not differ in the initial abilities of all evaluated cognitive functions such as MMSE, digit span, FIM cognition score, IQ of WAIS, AQ, and GDS (Table 1; Supplementary Table 1). Moreover, these clinical characteristics including baseline cognitive abilities and depressive symptoms were not different between the rTMS-treated and control groups at baseline for each hemispheric lesion (Supplementary Tables 2, 3). Regarding the existence of depression, 86.8% of the patients showed overt symptoms with a GDS score more than 10 (46) among all the recruited patients with left hemispheric stroke and 75% among all the recruited patients with right hemispheric stroke, without a difference between the two groups for each hemispheric lesion side. Through a medical review, 16 patients with left hemispheric lesion and 30 patients with right hemispheric lesion were reported to have taken antidepressants (sertraline 50 mg or escitalopram 5 mg or 10 mg/day) among total subjects. And all hospitalized patients, regardless of the rTMS treatment, medicated 5 mg of donepezil once a day.

**Table 1:** Demographic characteristics in total subjects (*n* = 133).

	**Total group**			**rTMS group**			**Control group**		
	**Total (*n* = 133)**	**Lt (*n* = 57)**	**Rt (*n* = 76)**	**Total (*n* = 49)**	**Lt (*n* = 22)**	**Rt (*n* = 27)**	**Total (*n* = 84)**	**Lt (*n* = 35)**	**Rt (*n* = 49)**
Age (years)	61.0 ± 13.8	60.4 ± 13.5	61.4 ± 14.1	60.4 ± 14.4	59.4 ± 13.5	61.2 ± 15.2	61.3 ± 13.5	61.1 ± 13.6	61.5 ± 13.6
									
Gender (*n*, %)									
Male	81 (60.9%)	38 (66.7%)	43 (56.6%)	28 (57.2%)	15 (68.2%)	13 (48.1%)	54 (64.3%)	23 (65.7%)	30 (61.2%)
Female	52 (39.1%)	19 (33.3%)	33 (43.4%)	21 (42.8%)	7 (31.8%)	14 (51.9%)	30 (35.7%)	12 (34.3%)	19 (38.7%)
Type of stroke (*n*, %)									
Infarction	60 (45.1%)	22 (38.6%)	38 (50.0%)	22 (44.9%)	9 (40.9%)	13 (48.1%)	38 (45.3%)	13 (37.1%)	25 (51.0%)
ICH	65 (48.9%)	32 (56.1%)	33 (43.4%)	22 (44.9%)	11 (50.0%)	11 (40.8%)	43 (51.2%)	20 (57.2%)	22 (44.9%)
SAH	8 (6.0%)	3 (5.3%)	5 (6.6%)	5 (10.2%)	5 (9.1%)	3 (11.1%)	3 (3.5%)	2 (5.7%)	2 (4.1%)
Post-stroke duration (day)	53.2 ± 18.1	54.1 ± 18.34	52.5 ± 18.1	49.8 ± 16.9	51.3 ± 14.7	48.6 ± 18.7	55.1 ± 18.7	55.9 ± 20.3	54.6 ± 17.6
Dominant hand									
Right/Left	133/0	57/0	76/0	49/0	22/0	27/0	84/0	35/0	49/0
									
Visual neglect	22 (16.5%)	1 (1.7%)	21 (27.63%)	8 (16.3%)	1 (4.5%)	7 (25.92%)	14 (16.6%)	0	14 (28.57%)
Aphasia	55 (41.3%)	37 (64.9%)	18 (23.6%)	22 (44.8%)	15 (68.1%)	7 (25.92%)	33 (39.2%)	22 (62.8%)	11 (22.4%)
K-MMSE	15.3 ± 7.5	12.8 ± 7.8^*^	17.1 ± 6.7	14.4 ± 7.7	12.6 ± 6.8	15.9 ± 8.2	15.7 ± 7.4	13.0 ± 8.5^*^	17.7 ± 5.8
FDS	4.3 ± 2.0 (114)	3.1 ± 2.5^*^ (46)	4.1 ± 2.1 (68)	4.0 ± 2.1 (48)	3.9 ± 2.2 (21)	4.1 ± 2.0 (27)	4.5 ± 1.9 (66)	3.8 ± 2.2^*^ (25)	5.0 ± 1.5 (41)
BDS	2.1 ± 1.4 (114)	1.4 ± 1.4^*^ (46)	2.2 ± 1.5 (68)	2.0 ± 1.2 (48)	1.9 ± 1.3 (21)	2.1 ± 1.3 (27)	2.2 ± 1.5 (66)	1.6 ± 1.5^*^ (25)	2.7 ± 1.4 (41)
FIM cog	18.1 ± 8.0 (116)	15.5 ± 6.5^*^ (52)	19.2 ± 9.4 (64)	17.5 ± 8.2 (44)	15.5 ± 5.5 (22)	19.5 ± 9.9 (22)	18.7 ± 7.9 (72)	15.5 ± 7.2^*^ (30)	21.0 ± 7.6 (42)
IQ of WAIS	62.4 ± 15.9 (108)	48.1 ± 27.3^*^ (47)	55.2 ± 26.3 (61)	59.6 ± 16.7 (41)	57.3 ± 19.0 (21)	62.1 ± 14.0 (20)	63.4 ± 15.1 (67)	59.2 ± 15.9 (26)	65.6 ± 14.1 (41)
AQ (%)	68.8 ± 31.1 (122)	49.3 ± 35.0^*^ (52)	74.4 ± 31.1 (70)	63.9 ± 33.1 (45)	53.0 ± 32.6^*^ (21)	73.4 ± 31.0 (24)	71.5 ± 29.9 (77)	54.7 ± 33.6^*^ (31)	82.8 ± 20.8 (46)
GDS	15.4 ± 8.3 (97)	13.3 ± 8.4^*^ (44)	17.0 ± 7.9 (53)	17.1 ± 9.9 (38)	15.4 ±10.5 (21)	19.1 ± 9.0 (17)	14.6 ± 7.0(59)	11.4 ± 4.5^*^ (23)	16.6 ± 7.2 (36)

* *P < 0.05 significantly lower in baseline comparison within group between right side and left side*.

*There were no differences between rTMS and control group in all compared variables for total subjects, left hemispheric lesion, and right hemispheric lesion patients (Supplementary Tables 1–3)*.

*rTMS, repetitive Transcranial Magnetic Stimulation; ICH, Intracranial hemorrhage; SAH, Subarachnoid hemorrhage; MMSE, Mini-Mental State Examination; FDS, Forward Digit span; BDS, Backward Digit Span; FIM cog, Cognitive Functional Independence Measure; WAIS, Wechsler Adult Intelligence Scale; GDS, Geriatric Depression Scale; AQ, Aphasia Quotient*.

*Age, post-stroke duration and evaluation scores were compared by independent t-test*.

*Patients with subarachnoid hemorrhage due to aneurysm rupture were included only for the cases whom were able to locate laterality of lesions*.

*(n) Number of patients evaluated, without remark all patients were evaluated*.

When the initial cognitive functions were compared between the left and right hemispheric lesions, all evaluated scores of cognition and affection, including MMSE, digit span, FIM cognition score, IQ of WAIS, AQ, and GDS, were poorer in the left hemispheric lesion group. Among the rTMS-treated group, the AQ of the left hemispheric lesion was lower than that of the right hemispheric lesion, indicating that the left lesion was more aphasic. In total subjects, 64.9 % of left hemispheric lesion and 23.7 % of right hemispheric patients presented aphasia, and there was no difference in the prevalence between the rTMS-treated and control groups. Among the control group, MMSE total, digit span scores, FIM cognition AQ, and GDS of patients with left hemispheric lesions were lower than those of patients with right hemispheric lesions (Table 1). These baseline differences show the typical characteristics of left brain lesions in cognition and speech impairments, as reported in previous reports (47, 48). As for visual neglect, 27.6 % of total right hemispheric lesion patients showed to have neglect, and there was no difference in the prevalence between the rTMS-treated and control groups (Table 1).

### Total Patient Analyses

#### Changes in Cognitive Measures and Depression in the rTMS and Control Groups

After 4 weeks, both the rTMS-treated group (*n* = 49) and the control group (*n* = 84) showed significant improvements in most of the assessments. Total MMSE and all of its subscores (orientation, registration, recall, attention/concentration, and language) were elevated (*P* < 0.001). Forward and backward digit span, FIM cognition score, AQ, IQ, and all of its subscores increased in both groups (*P* < 0.001). However, the GDS score showed amelioration of depression only in the rTMS-treated group (*P* < 0.001) and not in the control group (Table 2).

**Table 2:** Comparison of rehabilitation outcomes between rTMS and control groups (total subjects, *N* = 133).

	**rTMS group (total** ***n*** **=** **49)**		**Control group (total** ***n*** **=** **84)**		***P* value^†^ (between groups)**
	**Baseline**	**Follow-up**	**Baseline**	**Follow-up**	
MMSE	14.4 ± 7.7	**22.4** ±5.8^*^	15.7 ± 7.4	**20.3** ±7.8^*^	**0.001^††^**
Orientation	4.8 ± 2.8	**8.1** ±2.0^*^	5.0 ± 2.7	**7.4** ±2.9^*^	**0.037** ^†^
Registration	2.1 ± 1.3	**2.7** ±0.6^*^	2.4 ± 1.1	**2.6** ±0.8^*^	**0.024** ^†^
Recall	1.1 ± 1.4	**2.3** ±1.6^*^	0.9 ± 1.3	**1.6** ±1.5^*^	**0.014** ^†^
Attention/concentration	1.0 ± 1.3	**2.7** ±1.7^*^	1.1 ± 1.4	**1.8** ±1.7^*^	**<0.001^††^**
Language	5.0 ± 2.6	**6.6** ±1.8^*^	5.2 ± 2.5	**6.2** ±2.2^*^	0.043^*^
					
Digit span					
Forward	4.0 ± 2.1	**5.3** ±1.7^*^	4.5 ± 1.9	**5.0** ±1.7^*^	**<0.001^††^**
Backward	2.0 ± 1.2	**2.7** ±1.2^*^	2.2 ± 1.5	**2.7** ±1.4^*^	0.189
FIM					
Cognition	17.5 ± 8.2	**22.7** ±10.0^*^	18.7 ± 7.9	**20.8** ±8.3^*^	**<0.001^††^**
IQ	59.6 ± 16.7	**69.9** ±18.5^*^	63.4 ± 15.1	**71.4** ±15.9^*^	0.178
VCI	76.5 ± 23.7	**85.1** ±21.4^*^	82.3 ± 18.8	**89.5** ±18.4^*^	0.527
PRI	62.8 ± 18.9	**71.6** ±20.9^*^	64.3 ± 13.4	**72.9** ±16.5^*^	0.940
WMI	69.3 ± 77.1	**77.1** ±20.5^*^	72.6 ± 17.8	**80.0** ±18.0^*^	0.803
PSI	56.5 ± 13.7	**65.2** ±18.3^*^	60.5 ± 12.9	**65.9** ±16.0^*^	0.141
AQ	63.9 ± 33.1	**75.6** ±26.5^*^	71.5 ± 29.9	**79.1** ±24.8^*^	0.085
GDS	17.1 ± 9.9	**13.5** ±8.7^*^	14.6 ± 7.0	13.5 ± 7.8	0.060

* *Bold values; P < 0.05 significantly higher than baseline within each group comparison*.

*P value † compared the changes in each score from baseline to follow-up between two groups by independent t-test*.

† *P < 0.05*,

†† *P < 0.01*.

*All values are presented a mean ± standard deviation*.

*(n) Number of patients evaluated, without remark all patients were evaluated*.

*MMSE, Mini-Mental State Examination; FIM, Functional Independence Measure; IQ, Intellectual Quotient; VCI, Verbal Comprehension Index; PRI, Perceptual Reasoning Index; WMI, Working Memory Index; PSI, Processing Speed Index; AQ, Aphasia Quotient; GDS, Geriatric Depression Scale*.

#### Comparison of Outcomes Between the rTMS and Control Groups

In a comparison analysis between the two groups by change of each evaluation score, the rTMS-treated group showed bigger improvements in cognition including total MMSE and all of its subscores (*Ps* < 0.05), especially in the “attention/concentration” domain (*P* < 0.001), forward digit span (*P* < 0.001), and FIM cognition score (*P* < 0.001), compared with the control group. There was no significant difference in the backward digit span, IQ, and AQ. Changes in the GDS score showed a trait of better outcomes in depression in the rTMS-treated group than in the control group (*P* = 0.06) (Table 2).

### Analyses of Patients With Left Hemispheric Lesions

#### Changes in Cognitive Measures and Depression in the rTMS and Control Groups

The rTMS-treated group (*n* = 22) and control group (*n* = 35) showed improvements in all measured cognitive tests (*P* < 0.05). However, the GDS score showed amelioration of depression only in the rTMS-treated group (*P* = 0.001) and not in the control group (Table 3).

**Table 3:** Comparison of rehabilitation outcomes between rTMS and control groups (left hemispheric stroke subjects, *N* = 57).

	**rTMS group (total** ***n*** **=** **49)**		**Control group (total** ***n*** **=** **84)**		***P* value^†^ (between groups)**
	**Baseline**	**Follow-up**	**Baseline**	**Follow-up**	
MMSE	12.6 ± 6.8	**21.1** ±6.1^*^	13.0 ± 8.5	**17.6** ±9.2^*^	**0.004^††^**
Orientation	4.6 ± 2.7	**8.1** ±2.0^*^	4.1 ± 3.2	**6.7** ±3.7^*^	0.204
Registration	1.8 ± 1.4	**2.5** ±0.9^*^	1.9 ± 1.3	**2.4** ±1.0^*^	0.399
Recall	0.8 ± 1.0	**1.5** ±1.1^*^	0.5 ± 0.9	**1.0** ±1.0^*^	0.484
Attention/concentration	0.6 ± 0.9	**2.3** ±0.3^*^	0.8 ± 1.3	**1.5** ±1.7^*^	**0.016** ^†^
Language	4.3 ± 2.4	**6.1** ±2.1^*^	4.1 ± 2.5	**5.3** ±2.6^*^	0.263
					
Digit span					
Forward	3.9 ± 2.2	**5.0** ±2.0^*^	3.8 ± 2.2	**4.6** ±2.1^*^	0.306
Backward	1.9 ± 1.3	**2.3** ±1.2^*^	1.6 ± 1.5	**2.4** ±1.7^*^	0.228
FIM					
Cognition	15.5 ± 5.5	**20.9** ±5.8^*^	15.5 ± 7.2	**18.6** ±8.6^*^	**0.039** ^†^
IQ	57.3 ± 19.0	**68.0** ±21.6^*^	59.2 ± 15.9	**68.1** ±17.5^*^	0.485
VCI	69.8 ± 24.8	**79.6** ±24.2^*^	69.0 ± 23.0	**77.0** ±23.3^*^	0.683
PRI	65.0 ± 22.8	**72.4** ±24.7^*^	68.3 ± 15.3	**77.0** ±17.8^*^	0.696
WMI	64.9 ± 20.7	**73.1** ±22.9^*^	66.7 ± 17.4	**74.1** ±19.2^*^	0.720
PSI	55.5 ± 17.2	**64.3** ±22.4^*^	60.0 ± 12.6	**64.6** ±17.6^*^	0.248
AQ	53.0 ± 23.6	**74.5** ±23.8^*^	54.7 ± 23.6	**68.3** ±28.1^*^	0.098
GDS	15.4 ± 6.5	**9.8** ±5.9^*^	11.4 ± 4.5	11.1 ± 5.6	**0.002^††^**

* *Bold values; P < 0.05 significantly higher than baseline within each group comparison*.

*P value † compared the changes in each score from baseline to follow-up between two groups by independent t-test*.

† *P < 0.05*,

†† *P < 0.01*.

*All values are presented a mean ± standard deviation*.

*(n) Number of patients evaluated, without remark all patients were evaluated*.

*MMSE, Mini-Mental State Examination; FIM, Functional Independence Measure; IQ, Intellectual Quotient; VCI, Verbal Comprehension Index; PRI, Perceptual Reasoning Index; WMI, Working Memory Index; PSI, Processing Speed Index; AQ, Aphasia Quotient; GDS, Geriatric Depression Scale*.

#### Comparison of Outcomes Between rTMS and Control Groups

In the comparison analysis between the two groups, the rTMS-treated group showed greater improvements compared with the control group in total MMSE, “attention/concentration” subscore of MMSE, and FIM cognition (*P* < 0.05). Improvement in mood assessed using the GDS score indicated amelioration of depression induced by rTMS, with a significant difference between the two groups (*P* = 0.002) (Table 3).

### Analyses of Patients With Right Hemispheric Lesions

#### Changes in Cognitive Measures and Depression in the rTMS and Control Groups

The rTMS group (*n* = 27) showed improvements in all measured cognitive scores (*P* < 0.05). In contrast, the control group did not show significant increments in scores of “recall” subscore of MMES (*P* = 0.103) and backward digit span, although it may have marginal significance (*P* = 0.058). The control group also showed improvements in overall cognitive measures (*P* < 0.05). Both groups did not show changes in GDS scores (Table 4).

**Table 4:** Comparison of rehabilitation outcomes between rTMS and control groups (right hemispheric stroke subjects, *N* = 76).

	**rTMS group (total** ***n*** **=** **49)**		**Control group (total** ***n*** **=** **84)**		***P* value^†^ (between groups)**
	**Baseline**	**Follow-up**	**Baseline**	**Follow-up**	
MMSE	15.9 ± 8.2	**23.4** ±5.5^*^	17.7 ± 5.8	**22.2** ±5.9^*^	**0.003^††^**
Orientation	5.0 ± 2.9	**8.0** ±2.1^*^	5.7 ± 2.9	**8.0** ±2.1^*^	0.157
Registration	1.4 ± 0.5	**3.0** ±0.6^*^	1.2 ± 0.4	**2.0** ±0.7^*^	**0.009^††^**
Recall	2.3 ± 1.2	**2.8** ±1.4^*^	2.7 ± 1.7	2.8 ± 1.6	**0.013** ^†^
Attention/concentration	1.4 ± 0.5	**3.0** ±1.1^*^	1.2 ± 0.4	**2.0** ±1.1^*^	**0.008^††^**
Language	5.5 ± 2.6	**7.0** ±1.5^*^	6.1 ± 2.2	**6.8** ±1.7^*^	0.114
Digit span					
Forward	4.1 ± 1.0	**5.5** ±1.5^*^	5.0 ± 1.5	**5.2** ±1.49^*^	**0.004^††^**
Backward	2.1 ± 1.3	**3.0** ±1.1^*^	2.7 ± 1.4	2.9 ± 1.26	**0.015** ^†^
FIM					
cognition	19.5 ± 9.9	**23.5** ±12.8^*^	21.0 ± 7.6	**22.3** ±7.8^*^	**0.002^††^**
IQ	62.1 ± 14.0	**71.9** ±14.7^*^	65.6 ± 14.1	**73.2** ±14.7^*^	0.321
VCI	83.6 ± 20.9	**90.8** ±16.8^*^	89.0 ± 15.4	**95.6** ±16.3^*^	0.753
PRI	60.5 ± 13.8	**70.7** ±16.6^*^	61.7 ± 11.6	**70.4** ±15.2^*^	0.774
WMI	74.1 ± 17.3	**81.3** ±17.3^*^	76.4 ± 17.1	**83.7** ±16.3^*^	0.977
PSI	57.6 ± 9.1	**66.2** ±13.1^*^	60.8 ± 13.3	**66.8** ±15.1^*^	0.357
AQ	73.4 ± 31.0	**76.5** ±29.2^*^	82.8 ± 20.8	**86.5** ±19.5^*^	0.624
GDS	19.1 ± 9.0	18.0 ± 8.7	16.6 ± 7.2	15.0 ± 7.7	0.975

* *, Bold values; P < 0.05 significantly higher than baseline within each group comparison*.

*P value † compared the changes in each score from baseline to follow-up between two groups by independent t-test*.

† *P < 0.05*,

†† *P < 0.01*.

*All values are presented a mean ± standard deviation*.

*(n) Number of patients evaluated, without remark all patients were evaluated*.

*MMSE, Mini-Mental State Examination; FIM, Functional Independence Measure; IQ, Intellectual Quotient; VCI, Verbal Comprehension Index; PRI, Perceptual Reasoning Index; WMI, Working Memory Index; PSI, Processing Speed Index; AQ, Aphasia Quotient; GDS, Geriatric Depression Scale*.

#### Comparison of Outcomes Between the rTMS and Control Groups

In the comparison analysis between the two groups, the rTMS-treated group showed higher scores in total MMSE and three of its subscores: “registration,” “recall,” “attention/concentration,” both forward and backward digit span, and FIM cognition score (*P* < 0.05) compared with the control group (Table 4).

#### Side Effects

The medical records did not report any serious adverse events related to rTMS. Only three patients discontinued rTMS due to mild headache after their first treatment; thus, they were excluded from this analysis. None of the patients showed deterioration of function.

## Discussion

According to the results of the present retrospective study, high-frequency rTMS on the ipsilesional DLPFC might have beneficial effects on cognition in patients with stroke during their subacute phase when the patients are receiving intensive rehabilitation. Although the control group also showed improvement in cognitive function across the board, the rTMS-treated group showed remarkably better outcomes in cognition and mood recovery.

In comparison with the rTMS-treated group and controls without dividing lesions, the rTMS-treated group demonstrated efficacy in total MMSE and all subscores. Since it was invented as a screening tool for dementia, despite of its broad use, it is still controversial whether the MMSE is an appropriate tool for post-stroke patients. However, many previous literatures (49, 50) reported the MMSE to be sufficiently accurate as a screening tool for stroke patients. And it is obvious that MMSE is practically applicable in clinics for post-stroke patients. Among the subscores, greater improvement in the rTMS-treated group was evident in the “attention/concentration” domain.

In another attention representing test, the forward digit span also showed better outcomes in the rTMS-treated group. “Attention” is the most basic ability for further cognitive processes, and working memory is one of the most directly associated functions (51). Furthermore, the rTMS-treated group showed better outcomes in ADL by a higher gain of the FIM cognition score compared with the control group. Therefore, it seems that rTMS treatment exerted a therapeutic effect on attention, which led to better cognitive performance in the daily living of patients with stroke. There was no difference in changes in IQ between the groups with similarly increased scores during the study period. This may be because the Wechsler scale is not specifically suitable for the assessment of cognitive impairment in patients with stroke, especially to evaluate changes over a month (52). Nevertheless, WAIS-IV was adopted as a cognition scale because there is a lack of other quantitative scales to assess intelligence and sensitively catch cognitive changes in post-stroke patients. For depression, a marginally significant improvement in the rTMS-treated group was observed, which seems to be mostly beneficial for patients with left hemispheric stroke.

The most remarkable finding in this study was the differential responses in cognition and mood recovery by ipsilesional rTMS according to the hemispheric lesion side. In the left DLPFC rTMS-treated group, who had left hemispheric stroke, the significant items that showed beneficial effects of rTMS were total MMSE, only the “attention/concentration” subscore of MMSE, FIM cognition, and GDS. Although the aphasia might have affected other cognitive scales, it seems obvious that attention have attributed the most in cognitive improvement after rTMS. Meanwhile, the right rTMS-treated group, who had right hemispheric stroke, showed better outcomes than the control group in total MMSE, “registration,” “recall,” and “attention/concentration” subscores of MMSE, both forward and backward digit spans, and finally FIM cognition. According to clinical research, both DLPFC are well-acknowledged regions that implement various cognitive functions and attention control (15, 17, 21, 53). Through a comparison between two group analyses for each hemispheric lesion, ipsilesional DLPFC rTMS treatment in both hemispheres showed common effects on cognitive recovery, especially on attention in post-stroke patients, and the results of our study also support previous studies. The significantly higher increment of FIM cognition score in the rTMS-treated group of each hemispheric lesion seems to be meaningful because cognitive recovery has consequently led to improvement of ADL in the rTMS-treated patients.

In the present study, the most pronounced and specific effect of left DLPFC rTMS was amelioration of depression in patients with left hemispheric stroke. Recent studies have shown that high-frequency rTMS over the left DLPFC has a beneficial effect on refractory major depression and post-stroke depression (54). In this study, we also found a significant reduction in GDS score after rTMS treatment over the left DLPFC, while in the right DLPFC group, there was no effect on depression (55). Our retrospective analysis could provide basis for further prospective studies with new protocol of rTMS treatment depending on the laterality of the lesion in post-stroke patients.

In the right DLPFC rTMS-treated group, having right hemispheric lesions, total MMSE, and the subscores of “registration,” “recall,” and “attention/concentration” showed significant findings of better outcome. This seems to be a meaningful result considering the common tendency of attention deficits in patients with right hemisphere lesions (56). In contrast to the left DLPFC rTMS-treated group who showed improvement only in the forward digit span, the right side rTMS-treated group showed improvement in the backward digit span, which indicates amelioration of working memory. Recovery of cognitive function after stroke, particularly attention and working memory, is directly related to the successful prognosis of cognition (57). Among working memory, verbal memory is measured by typical retention tasks such as forward and backward digit span tests (58). Our results on digit span score improvements in the right DLPFC-treated group are consistent with previous findings that reported activation in the right DLPFC rather than in the left DLPFC in verbal episodic retrieval (59–61). In a more recent study, a neural mechanism involving the DLPFC in proactive and reactive control as a key regulator was suggested with a more specific role of the left DLPFC in proactive control and the implication of the right DLPFC in reactive control related to retrieval of verbal information (59). Our results of increments in MMSE subscores, registration and recall progression only by right DLPFC rTMS treatment are consistent with previous research about the right DLPFC, which is responsible for verbal retrieval and short-term memory such as reactive control.

Since this research was not prospectively conducted, there are several weak points. First of all, our results have inevitable limitations as a retrospective study, which is also prone to selection bias. To reduce the bias as much as possible, control group patients were enrolled without exception who were admitted at the same time and criteria for inclusion and exclusion were the same for each group. Only different points between the groups were treatment of rTMS ≥ 5 times over ipsilesional DLPFC or none rTMS. And no significant differences were found in basal demographic characteristics between the groups regarding age, sex, type of stroke, and post-stroke duration. Even though, retrospective analysis cannot be free from the observer effect and unrecognized confounder factors. Second, the number of rTMS treatment therapies varied, and the range of cognitive impairment of the patients was wide. While the baseline functional scores were not different between the rTMS and control groups, it could not assure the homogeneity of the two compared groups. There were differences in cognitive function between the left and right hemispheric lesion patients within each treatment group, and the differences were more prevalent in the control group. This seemed to be caused by the differences in the number of enrolled patients in the groups. Even though significant results appeared during the one-month follow-up, long-term therapeutic effects are needed through follow-up studies, and a randomized controlled prospective study is required. It also seems that visual neglect and aphasia need to be approached by subdividing the group who have the problem for understanding the efficacy mechanism on cognition. For example, the improvements in right hemispheric lesion group needs to be interpreted through further study especially considering possible link with unilateral neglect which was not fully addressed in this study. The effect of contralesional rTMS on the DLPFC is also needed.

This is the first attempt to determine the effects of rTMS, which can vary depending on the treatment side of the DLPFC. Although further functional and structural studies of rTMS on the right and left DLPFC separately are needed, this study could support the therapeutic potential of rTMS over the ipsilesional DLPFC on cognitive restoration and alleviation of post-stroke depression.

In conclusion, high-frequency rTMS over the ipsilesional DLPFC showed beneficial effects on cognition recovery, and those who received rTMS over the left DLPFC had improved depression in patients with cerebral hemispheric stroke during the subacute phase.

## Data Availability Statement

The raw data supporting the conclusions of this article will be made available by the authors, without undue reservation.

## Ethics Statement

The studies involving human participants were reviewed and approved by Cha University Institutional Review Board (IRB file No: 2017-09-060). Written informed consent for participation was not required for this study in accordance with the national legislation and the institutional requirements.

## Author Contributions

All authors listed have made a substantial, direct, and intellectual contribution to the work and approved it for publication.

## Funding

This research was supported by a grant from the Korea Health Technology R&D Project through the Korea Health Industry Development Institute (KHIDI), funded by the Ministry of Health and Welfare, Republic of Korea (Grant Number: HI16C1559) and Institute of Information & Communications Technology Planning and Evaluation (IITP) grant funded by the Korea Government (MSIP) (2021-0-00742 Development of Core Technology for Whole-body Medical Twin).

## Conflict of Interest

The authors declare that the research was conducted in the absence of any commercial or financial relationships that could be construed as a potential conflict of interest.

## Publisher's Note

All claims expressed in this article are solely those of the authors and do not necessarily represent those of their affiliated organizations, or those of the publisher, the editors and the reviewers. Any product that may be evaluated in this article, or claim that may be made by its manufacturer, is not guaranteed or endorsed by the publisher.
