# Supplementary material for: Effect of Cognition Recovery by Repetitive Transcranial Magnetic Stimulation on Ipsilesional Dorsolateral Prefrontal Cortex in Subacute Stroke Patients
Source: Front Neurol. 2022 Jan 31;13:823108. doi: 10.3389/fneur.2022.823108 (PMC8848770; doi:10.3389/fneur.2022.823108)
Supplement: Supplementary file 1 [file Table_1.docx]

**[ Supplementary Table 1 ]**

**Demographic Characteristics in Total Subjects (N =133)**

|  | rTMS group (*n*=49) | | |  |  | Control group (*n* =84) | | | | |  | | *Between*  *Total group*  *P* value^†^ | |
| --- | --- | --- | --- | --- | --- | --- | --- | --- | --- | --- | --- | --- | --- | --- |
|  | Total (*n*=49) | Lt (*n*=22) | Rt (*n*=27) | *Lt & Rt*  *P* value* |  | Total (*n*=84) | Lt (*n*=35) | Rt (*n*=49) | *Lt & Rt*  *P* value* |  | |  | |  |
| Age (years) | 60.4 ± 14.4 | 59.40 ± 13.57 | 61.25 ± 15.26 | 0.660 |  | 61.3 ± 13.5 | 61.14 ± 13.61 | 61.57 ± 13.62 | 0.887 |  | | 0.943 | |  |
|  |  |  |  |  |  |  |  |  |  |  | |  | |  |
| Gender (n, %) |  |  |  |  |  |  |  |  |  |  | |  | |  |
| Male | 28 (57.2%) | 15 (68.2%) | 13 (48.1%) |  |  | 54 (64.3%) | 23 (65.7%) | 30 (61.2%) |  |  | |  | |  |
| Female | 21 (42.8%) | 7 (31.8%) | 14 (51.9%) |  |  | 30 (35.7%) | 12 (34.3%) | 19 (38.7%) |  |  | |  | |  |
|  |  |  |  |  |  |  |  |  |  |  | |  | |  |
| Type of stroke (n, %) |  |  |  |  |  |  |  |  |  |  | |  | |  |
| Infarction | 22 (44.9%) | 9 (40.9%) | 13 (48.1%) |  |  | 38 (45.3%) | 13 (37.1%) | 25 (51.0%) |  |  | |  | |  |
| ICH | 22 (44.9%) | 11 (50.0%) | 11 (40.8%) |  |  | 43 (51.2%) | 20 (57.2%) | 22 (44.9%) |  |  | |  | |  |
| SAH | 5 (10.2%) | 5 (9.1%) | 3 (11.1%) |  |  | 3 (3.5%) | 2 (5.7%) | 2 (4.1%) |  |  | |  | |  |
|  |  |  |  |  |  |  |  |  |  |  | |  | |  |
| Post-stroke  duration (day) | 36.6 ± 22.1 | 33.6 ± 21.4 | 36.2 ± 21.4 | 0.432 |  | 39.1 ± 25.0 | 37.1 ± 23.0 | 38.1 ± 24.0 | 0.361 |  | | 0.532 | |  |
|  |  |  |  |  |  |  |  |  |  |  | |  | |  |
| Premorbid  dominant hand |  |  |  |  |  |  |  |  |  |  | |  | |  |
| Right / Left | 49 / 0 | 22 / 0 | 27 / 0 |  |  | 84 / 0 | 35 / 0 | 49 / 0 |  |  | |  | |  |
|  |  |  |  |  |  |  |  |  |  |  | |  | |  |
| K-MMSE - total | 14.46 ± 7.76 | 12.63 ± 6.85 | 15.96 ± 8.25 | 0.137 |  | 15.78 ± 7.43 | 13.05 ± 8.58* | 17.73 ± 5.84 | **0.004*** |  | | 0.34 | |  |
| Digit span – forward | 4.02 ± 2.12 (48) | 3.90 ± 2.23 (21) | 4.11 ± 2.08 (27) | 0.743 |  | 4.57 ± 1.90 (66) | 3.88 ± 2.26 (25)* | 5.00 ± 1.53 (41) | **0.019*** |  | | 0.15 | |  |
| Digit span – backward | 2.06 ± 1.29 (48) | 1.90 ± 1.30 (21) | 2.18 ± 1.30 (27) | 0.463 |  | 2.28 ± 1.56 (66) | 1.60 ± 1.50 (25)* | 2.70 ± 1.47 (41) | **0.017*** |  | | 0.41 | |  |
| FIM cognition score | 17.56 ± 8.20 (44) | 15.54 ± 5.52 (22) | 19.59 ± 9.93 (22) | 0.145 |  | 18.75 ± 7.92 (72) | 15.56 ± 7.26 (30)* | 21.02 ± 7.66 (42) | **0.006*** |  | | 0.44 | |  |
| IQ of WAIS | 59.68 ± 16.77 (41) | 57.33 ± 19.07 (21) | 62.15 ± 14.02 (20) | 0.212 |  | 63.44 ± 15.19 (67) | 59.26 ± 15.93 (26) | 65.62 ± 14.11 (41) | 0.090 |  | | 0.17 | |  |
| Aphasia quotient (%) | 63.91 ± 33.10 (45) | 53.02 ± 32.65 (21) | 73.43 ± 31.09 (24) | **0.038*** |  | 71.55 ± 29.93 (77) | 54.74 ± 33.65 (31)* | 82.87 ± 20.80 (46) | **0.000*** |  | | 0.20 | |  |
| GDS dep | 17.10 ± 9.93 (38) | 15.42 ±10.53 (21) | 19.17 ± 9.01 (17) | 0.262 |  | 14.61 ± 7.05(59) | 11.43 ± 4.54 (23)* | 16.63 ± 7.22 (36) | **0.005*** |  | | 0.15 | |  |

*; *P*<0.05 significantly lower in baseline comparison within group between right side and left side.

There were no differences between rTMS and control group in all compared variables for total subjects, left hemispheric lesion, and right hemispheric lesion patients.

rTMS; repetitive Transcranial Magnetic Stimulation, ICH; Intracranial hemorrhage, SAH; Subarachnoid hemorrhage, MMSE; Mini-Mental State Examination, FIM; Functional Independence Measure, WAIS; Wechsler Adult Intelligence Scale, GDS; Geriatric depression scale, AQ; Aphasia Quotient.

Age, post-stroke duration and evaluation scores were compared by independent t-test.

(n) Number of patients evaluated, without remark all patients were evaluated.
