# Supplementary material for: Effect of Cognition Recovery by Repetitive Transcranial Magnetic Stimulation on Ipsilesional Dorsolateral Prefrontal Cortex in Subacute Stroke Patients
Source: Front Neurol. 2022 Jan 31;13:823108. doi: 10.3389/fneur.2022.823108 (PMC8848770; doi:10.3389/fneur.2022.823108)
Supplement: Supplementary file 2 [file Table_2.docx]

**[ Supplementary Table 2 ]**

|  | rTMS group  (*n* =22) | Control group  (*n* =35) | *P*-value |
| --- | --- | --- | --- |
| Age (years, mean ± standard deviation) | 59.40 ± 13.57 | 61.14 ± 13.61 | 0.64 |
| Gender (n, %) |  |  |  |
| Male | 15 (68.2%) | 23 (65.7%) |  |
| Female | 7 (31.8%) | 12 (34.3%) |  |
| Type of stroke (n, %) |  |  |  |
| Cerebral infarction | 9 (40.9%) | 13 (37.1%) |  |
| Intracranial hemorrhage | 11 (50.0%) | 20 (57.2%) |  |
| Subarachnoid hemorrhage | 5 (9.1%) | 2 (5.7%) |  |
| Post-stroke duration  (day, mean ± standard deviation) | 33.6 ± 21.4 | 37.1 ± 23.0 | 0.42 |
| Premorbid dominant hand |  |  |  |
| Right / Left | 22 / 0 | 35 / 0 |  |
| K-MMSE - total | 12.63 ± 6.85 | 13.05 ± 8.58 | 0.83 |
| Digit span - forward | 3.90 ± 2.23 (21) | 3.88 ± 2.26 (25) | 0.30 |
| Digit span - backward | 1.90 ± 1.30 (21) | 1.60 ± 1.50 (25) | 0.31 |
| Functional Independence Measure |  |  |  |
| Cognition score | 15.54 ± 5.52 (22) | 15.56 ± 7.26 (30) | 0.99 |
| Intellectual quotient of WAIS | 57.33 ± 19.07 (21) | 59.26 ± 15.93 (26) | 0.70 |
| Aphasia quotient (%) | 53.02 ± 32.65 (21) | 54.74 ± 33.65 (31) | 0.85 |
| Geriatric Depression Scale | 15.42 ±10.53 (21) | 11.43 ± 4.54 (23) | 0.81 |

**Demographic Characteristics in Left Hemispheric Lesion Subjects (N =57)**

rTMS; repetitive Transcranial Magnetic Stimulation, ICH; Intracranial hemorrhage, SAH; Subarachnoid hemorrhage, MMSE; Mini-Mental State Examination, FIM; Functional Independence Measure, WAIS; Wechsler Adult Intelligence Scale, GDS; Geriatric depression scale, AQ; Aphasia Quotient.

Age, post-stroke duration and evaluation scores were compared by independent t-test.

(n) Number of patients evaluated, without remark all patients were evaluated.
