# Supplementary material for: Effect of Cognition Recovery by Repetitive Transcranial Magnetic Stimulation on Ipsilesional Dorsolateral Prefrontal Cortex in Subacute Stroke Patients
Source: Front Neurol. 2022 Jan 31;13:823108. doi: 10.3389/fneur.2022.823108 (PMC8848770; doi:10.3389/fneur.2022.823108)
Supplement: Supplementary file 3 [file Table_3.docx]

**[ Supplementary Table 3 ]**

**Demographic Characteristics in Right Hemispheric Lesion Subjects (N =76)**

|  | rTMS group  (*n* = 27) | Control group  (*n* = 49) | *P*-value |
| --- | --- | --- | --- |
| Age (years, mean ± standard deviation) | 61.25 ± 15.26 | 61.57 ± 13.62 | 0.93 |
| Gender (n, %) |  |  |  |
| Male | 13 (48.1%) | 30 (61.2%) |  |
| Female | 14 (51.9%) | 19 (38.7%) |  |
| Type of stroke (n, %) |  |  |  |
| Cerebral infarction | 13 (48.1%) | 25 (51.0%) |  |
| Intracranial hemorrhage | 11 (40.8%) | 22 (44.9%) |  |
| Subarachnoid hemorrhage | 3 (11.1%) | 2 (4.1%) |  |
| Post-stroke duration  (day, mean ± standard deviation) | 36.2 ± 21.4 | 38.1 ± 24.0 | 0.73 |
| Premorbid dominant hand |  |  |  |
| Right / Left | 27 / 0 | 49 / 0 |  |
| K-MMSE - total | 15.96 ± 8.25 | 17.73 ± 5.84 | 0.28 |
| Digit span - forward | 4.11 ± 2.08 (27) | 5.00 ± 1.53 (41) | 0.30 |
| Digit span - backward | 2.18 ± 1.30 (27) | 2.70 ± 1.47 (41) | 0.31 |
| Functional Independence Measure |  |  |  |
| Cognition score | 19.59 ± 9.93 (22) | 21.02 ± 7.66 (42) | 0.51 |
| Intellectual quotient of WAIS | 62.15 ± 14.02 (20) | 65.62 ± 14.11 (41) | 0.64 |
| Aphasia quotient (%) | 73.43 ± 31.09 (24) | 82.87 ± 20.80 (46) | 0.13 |
| Geriatric Depression Scale | 19.17 ± 9.01 (17) | 16.63 ± 7.22 (36) | 0.24 |

rTMS; repetitive Transcranial Magnetic Stimulation, ICH; Intracranial hemorrhage, SAH; Subarachnoid hemorrhage, MMSE; Mini-Mental State Examination, FIM; Functional Independence Measure, WAIS; Wechsler Adult Intelligence Scale, GDS; Geriatric depression scale, AQ; Aphasia Quotient.

Age, post-stroke duration and evaluation scores were compared by independent t-test.

(n) Number of patients evaluated, without remark all patients were evaluated.
